# E-Band InAs Quantum Dot Micro-Disk Laser with Metamorphic InGaAs Layers Grown on GaAs/Si (001) Substrate

**DOI:** 10.3390/ma17081916

**Published:** 2024-04-21

**Authors:** Wenqian Liang, Wenqi Wei, Dong Han, Ming Ming, Jieyin Zhang, Zihao Wang, Xinding Zhang, Ting Wang, Jianjun Zhang

**Affiliations:** 1 School of Physics, South China Normal University, Guangzhou 510631, China; 2 Songshan Lake Materials Laboratory, Dongguan 523808, China; weiwenqi@sslab.org.cn (W.W.);; 3 Beijing National Laboratory for Condensed Matter Physics, Institute of Physics, Chinese Academy of Sciences, Beijing 100190, China

**Keywords:** III-V on silicon, micro-disk lasers, E-band quantum dots, photonic integrated circuits, molecular beam epitaxy

## Abstract

The direct growth of III-V quantum dot (QD) lasers on silicon substrate has been rapidly developing over the past decade and has been recognized as a promising method for achieving on-chip light sources in photonic integrated circuits (PICs). Up to date, O- and C/L-bands InAs QD lasers on Si have been extensively investigated, but as an extended telecommunication wavelength, the E-band QD lasers directly grown on Si substrates are not available yet. Here, we demonstrate the first E-band (1365 nm) InAs QD micro-disk lasers epitaxially grown on Si (001) substrates by using a III-V/IV hybrid dual-chamber molecular beam epitaxy (MBE) system. The micro-disk laser device on Si was characterized with an optical threshold power of 0.424 mW and quality factor (Q) of 1727.2 at 200 K. The results presented here indicate a path to on-chip silicon photonic telecom-transmitters.

## 1. Introduction

The heterogeneous integration of III-V functional devices on silicon substrates is an important research field in future applications of information and communication. With the rapid development of silicon photonics, III-V quantum dot (QD) lasers integrated on CMOS-compatible silicon substrates have been becoming an essential component for photonic integrated chips (PICs) [1,2,3,4]. Over the past decade, a lot of research related to directly epitaxial growing III-V QD lasers on silicon and SOI substrates has been demonstrated, such as Fabry–Perot (FP) lasers [5,6,7,8,9,10,11], distributed feedback (DFB) lasers [12,13,14,15] and micro-cavity lasers [16,17,18,19,20]. However, almost all III-V lasers involve the O-band (original band: 1260–1360 nm) or C-band (conventional band: 1530–1565 nm) wavelengths, which are the low-loss wavelength regions used in traditional optical fibers for wavelength division multiplexing (WDM). In order to extend the optical communication window, the E-band (1360–1460 nm) wavelength has also been developed and used in optical fibers (ITU-T G.652.D) [21], which are fabricated by the dehydration process to reduce the relatively high loss from water absorption [22]. In previous research, several attempts have been made towards E-band III-V lasers, such as InGaAsP multi-quantum-well (MQW) lasers on InP [23], GaInNAs MQW lasers on GaAs [24] and InAs QD lasers on metamorphic InGaAs [25], but direct epitaxially grown E-band III-V lasers on Si substrates have not been reported yet. Additionally, microcavity lasers own unique advantages such as a small footprint, low power consumption and a high-quality (Q) factor, which can be used as compact on-chip light sources in PICs or sensing techniques.

In this work, we demonstrated direct epitaxially grown E-band InAs/InGaAs QD micro-disk lasers on Si (001) substrates. The step-graded metamorphic InGaAs buffer layers are implemented here to extend the emission wavelength of InAs QDs to the E-band [26,27,28,29], while the QD properties are also characterized by atomic force microscopy (AFM), cross-sectional transmission electron microscopy (TEM) and photoluminescence (PL) measurements. Furthermore, the fabricated InAs/InGaAs QD micro-disk laser on Si with a diameter of 4 μm is measured with an emission wavelength of 1364.5 nm and a threshold power of 0.424 mW. The results presented here pave a promising way for the epitaxial integration of III-V light sources on a silicon photonic platform.

## 2. Experimental Details

### 2.1. Materials’ Growth

**Figure 1:**
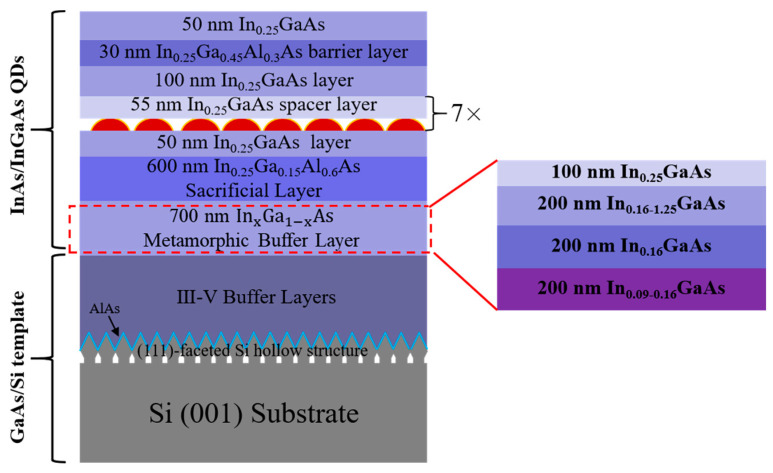
Schematic diagram of the E-band InAs QD micro-disk laser sample epitaxially grown on GaAs/Si (001) template with a 7-layer InAs/InGaAs QDs as active region. The right diagram shows the detailed structure of InGaAs metamorphic buffer layer grown by step-graded method.

Epitaxial growth of the sample was performed by a solid-source III-V/IV hybrid dual-chamber molecular beam epitaxy (MBE) system. A standard 8-inch Si (001) substrate was patterned with U-shaped grating structures along the [110] direction in CMOS process line. The U-shaped gratings with a period of 360 nm and a ridge width of approximately 140 nm were fabricated by deep ultraviolet (DUV) photolithography and dry etching process. After immersion in diluted hydrofluoric acid (HF) solution to remove the native oxide layer, the patterned Si (001) substrate was loaded in MBE chamber and the silicon homoepitaxial growth was conducted to construct (111)-faceted silicon hollow sawtooth structures on the U-shaped gratings. Then, the (111)-faceted silicon hollow substrate was transferred into the III-V MBE chamber for in situ III-V hybrid growth. As the schematic diagram shows in Figure 1, approximately 2100 nm thick III-V buffer layers were grown to achieve a smooth GaAs layer with low defect density on Si (001) substrate.

Here, a 10 nm AlAs nucleation layer and a 30 nm GaAs low-temperature buffer layer were first deposited at 380 °C to form a high-quality interface at Si (111) facets, as shown in Figure 1. The III-V buffer layers mainly consisted of 2 periods of InGaAs/GaAs and 2 repeats of InAlAs/GaAs quantum wells (QWs), both of which acted as dislocation filter layers (DFLs) to reduce the threading dislocation density (TDD) to the order of $10^{6}/{cm}^{2}$ [8,26,30]. Each of the DFLs included 5 periods of 10 nm-InGa(Al)As/10 nm-GaAs and 200 nm GaAs spacer layer. Lastly, the GaAs/AlGaAs superlattices (SLs) were also deposited to smooth the surface of the III-V buffer layers. The structural and growth details of the 2100 nm III-V buffer layers on Si can be found in our previous research [26,30].

To extend the emission wavelength of InAs QDs, step-graded metamorphic InGaAs buffer layers were introduced, as described in the earlier work [16,26]. Figure 1 schematically shows the whole structure of the E-band InAs QD micro-disk laser sample. Here, in order to achieve the E-band emission, an InGaAs layer with 25% indium content was grown on a 700 nm thick InGaAs metamorphic buffer layer grown by the step-graded epitaxial growth method ranging from ${In}_{0.09}{Ga}_{0.91}As$ to ${In}_{0.25}{Ga}_{0.75}As$. The detailed structure of the metamorphic buffer is also shown in Figure 1. Each layer of metamorphic InGaAs was 200 nm and grown at a low temperature of 380 °C to suppress the lattice-misfit-induced defects. Then, the thermal cycle annealing (TCA) process was introduced at an optimum temperature of 510 °C for high crystal quality and flat InGaAs layers [16]. Seven layers of InAs/${In}_{0.25}{Ga}_{0.75}As$ QDs as active region were grown on the 600 nm thick ${In}_{0.25}{Ga}_{0.15}{Al}_{0.6}As$ sacrificial layer. Each layer of the InAs QDs consisted of 3.1 monolayer (ML) InAs grown at 445 °C, which was capped by a 10 nm low-temperature ${In}_{0.25}{Ga}_{0.75}As$ layer (445 °C) and separated by a 45 nm ${In}_{0.25}{Ga}_{0.75}As$ spacer layer grown at higher temperature of 500 °C. The whole InAs/InGaAs QD active region was sandwiched by 50 nm and 100 nm lower and upper InGaAs buffer layers, respectively. A 30 nm ${In}_{0.25}{Ga}_{0.45}{Al}_{0.3}As$ layer was also grown as a barrier layer to increase the quantum efficiency of the lasers. Lastly, a 50 nm ${In}_{0.25}{Ga}_{0.75}As$ buffer layer was grown to achieve the smooth sample surface.

### 2.2. Material Characterizations

**Figure 2:**
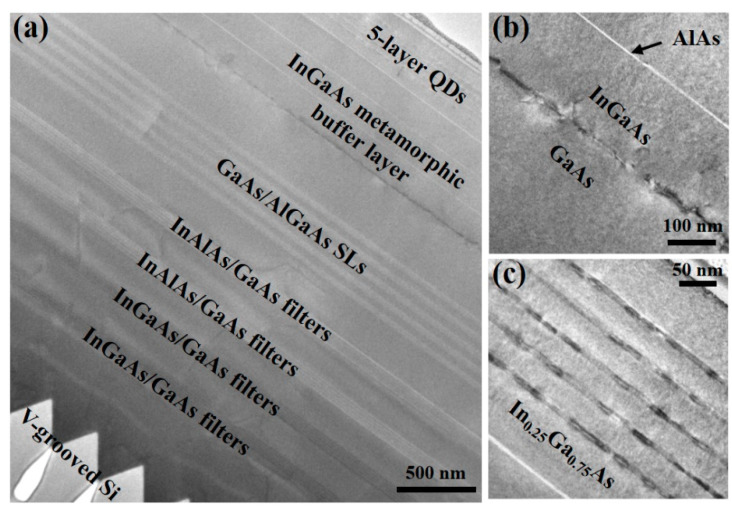
(**a**) Bright-field cross-sectional TEM image of a five-layer of E-band InAs/InGaAs QDs grown on InGaAs/GaAs/Si substrate. (**b**,**c**) show the zoomed-in TEM images at the InGaAs/GaAs interface and InAs QD active region, respectively.

A sample with a five-layer of E-band InAs/${In}_{0.25}{Ga}_{0.75}As$ QD active region and surface InAs QDs was grown on GaAs/Si substrate for material characterizations. The bright-field cross-sectional TEM measurements of the sample were conducted to characterize the crystal quality, as shown in Figure 2.

Benefiting from the homoepitaxially formed (111)-faceted sawtooth structures and the InGa(Al)As/GaAs DFLs, the defects were mostly confined to the GaAs/Si interface and III-V buffer region, without propagating into the upper region shown in Figure 2a. Smooth GaAs surface with approximately 1 nm roughness and $5\times 10^{6}/{cm}^{2}$ TDD can be obtained on these Si (001) substrates [26]. Figure 2b shows the zoomed-in TEM image of the interface between InGaAs metamorphic buffer layer and GaAs layer, which indicates that most of the lattice-mismatch-induced defects are confined to the interface due to the low-temperature growth method at 380 °C. The 3 nm thick AlAs layers between InGaAs metamorphic buffer layers can be also obviously distinguished due to the SEM contrast, which were grown to prevent the indium desorption at a high annealing temperature of 510 °C. Figure 2c shows the zoomed-in TEM image of the five layers of InAs/InGaAs QDs, and no obvious defects are observed in the active region.

**Figure 3:**
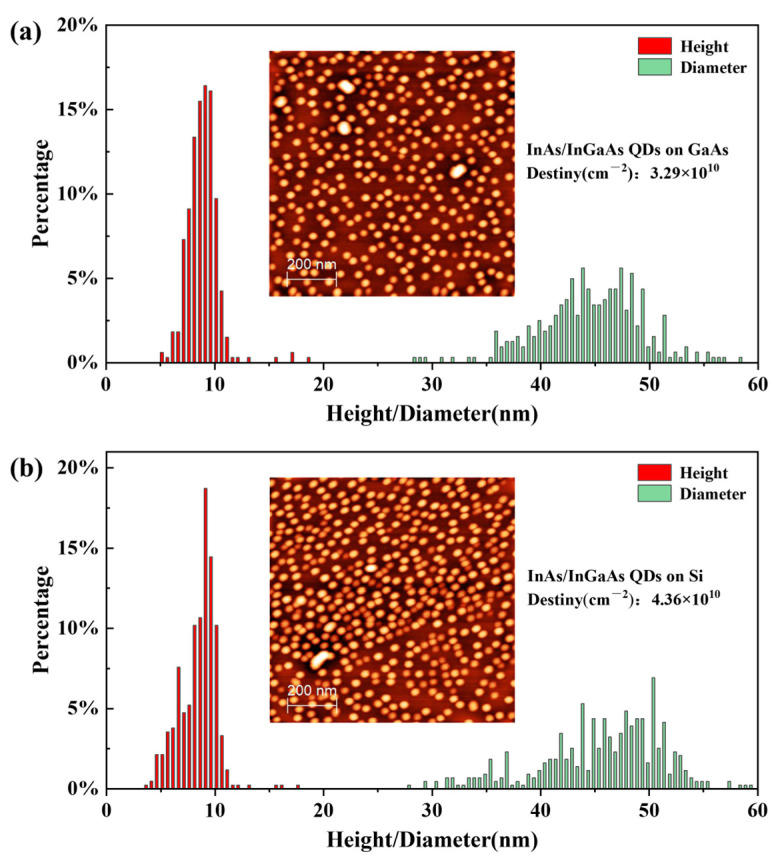
Diameter and height histograms of the surface InAs/InGaAs QDs on (**a**) GaAs (001) substrate and (**b**) GaAs/Si (001) substrate, respectively. Insets in (**a**,**b**) show the $1\times 1\;{\mu m}^{2}$ AFM images of the surface InAs/InGaAs QDs on these two substrates.

To accurately characterize the surface morphology of InAs/${In}_{0.25}{Ga}_{0.75}As$ QDs, AFM measurements of the surface QDs were conducted by a Bruker Dimension Icon instrument with a silicon tip coated with aluminum (Model: RTESPA-150) in the tapping mode on both GaAs (001) and GaAs/Si (001) substrates. Figure 3a,b show the diameter and height histograms of the surface InAs QDs on GaAs (001) and GaAs/Si (001) substrates, respectively.

**Figure 4:**
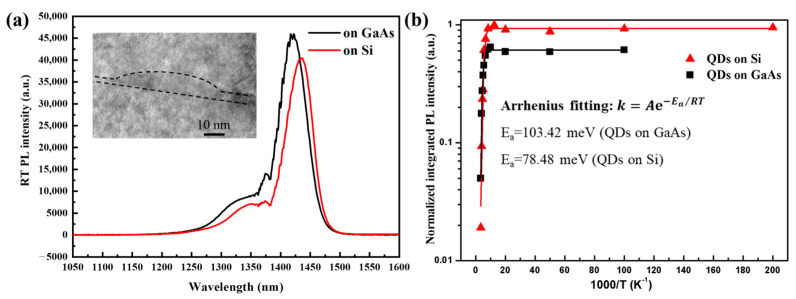
(**a**) Room-temperature PL spectra of InAs/InGaAs QDs on GaAs (001) and GaAs/Si (001) substrates, respectively. Inset: the cross-sectional TEM image of a buried InAs/InGaAs QD on InGaAs/GaAs/Si (001) substrate. (**b**) Arrhenius plots of temperature-dependent IPLI of the InAs QDs on GaAs and Si, respectively.

The insets show the $1\times 1\;{\mu m}^{2}$ AFM images of the InAs/InGaAs QDs on these two substrates with dot densities of $3.29\times 10^{10}/{cm}^{2}$ and $4.36\times 10^{10}/{cm}^{2}$, respectively. The average height and diameter of the QDs on GaAs (001) are 9.001 nm and 44.835 nm with the mean square error (MSE) of 1.483 nm and 5.753 nm, respectively. As a comparison, the average height and diameter of the QDs on GaAs/Si (001) substrate were also analyzed with values of 8.682 nm (MSE: 1.723 nm) and 45.728 nm (MSE: 7.409 nm), which are similar to those on GaAs (001) substrate. Additionally, a slightly elongated shape of the QDs on Si (001) substrate can be observed form the AFM image, which is caused by the unevenness of the InGaAs/GaAs/Si surface [16,26]. Compared with the O-band InAs/GaAs QDs reported in previous work [26], the E-band InAs/InGaAs QDs own larger sizes due to the smaller mismatch between InAs QD and InGaAs layers than that between InAs QD and GaAs layer. Moreover, the inset in Figure 4a shows the cross-sectional TEM image of a buried InAs/InGaAs QD, indicating a 7.5 nm height and 50 nm diameter.

In Figure 4a, room-temperature (RT) PL spectra of the InAs/InGaAs QDs on both GaAs (001) and Si (001) substrates are measured with a continuous-wave (CW) 532 nm solid-state pumping laser. E-band (1434.7 nm) PL peak emission with a full width at half maximum (FWHM) of 31.4 meV can be observed from the InAs/InGaAs QDs on Si (001). For InAs/InGaAs QDs on GaAs, the PL central wavelength and FWHM are 1421 nm and 33.5 meV, respectively. As shown in Figure 4a, a small shoulder can be distinguished from both of the spectra, which arises from the excited state of the QDs. Furthermore, the obvious noise-like PL spectra around 1400 nm can be also observed, which are caused by the defect of the gratings with the grating density of 600 grooves/mm used in the HORIBA monochromator for PL collection [16]. Moreover, the PL peak intensity of InAs/InGaAs QDs on Si substrate is about 87% of that on GaAs substrate, indicating that the Si-based InAs QDs own good QD quality and uniformity such as those on GaAs substrate. Additionally, more optical properties of the InAs QDs grown on these two substrates were investigated by the temperature-dependent PL measurements. Figure 4b shows the normalized integrated PL intensity (IPLI) plots of the two samples with a reciprocal temperature as the abscissa. The black and red curves are the Arrhenius fitting results of the experimental data by using the formula $k=Ae^{-E_{a}/RT}$ [31]. With the increase in temperature, the decrease in IPLI can be observed from the plot due to the thermal escape of the carriers from the ground state of QDs to surrounding layers. Here, the thermal activation energies ($E_{a}$) of the two samples can be calculated as the values of 103.42 meV (standard error: 6.27) and 78.48 meV (standard error: 2.29), respectively, for the InAs QDs on GaAs (001) and Si (001) substrates, which are almost the same as the values reported previously [26]. For the sample on Si substrate, the thermal activation energy of 78.48 meV is significantly lower than that on GaAs substrate, which is probably due to increased non-radiative recombination from the defects on GaAs/Si platform than that on native GaAs (001) substrate.

### 2.3. Device Fabrication

**Figure 5:**
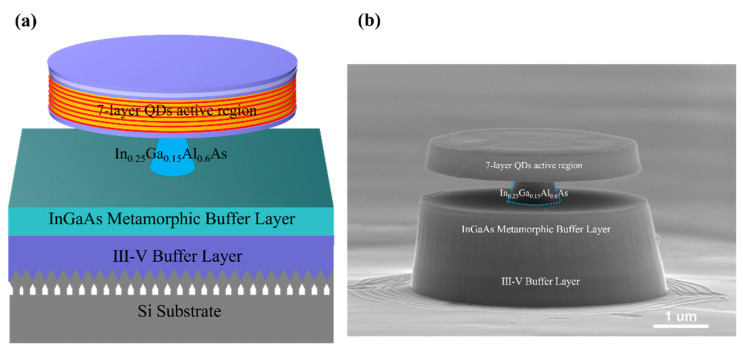
(**a**) Schematic diagram of an E-band micro-disk laser on GaAs/Si (001) substrate with seven layers of InAs/InGaAs QDs as active region and 600 nm InGaAlAs sacrificial layer. (**b**) SEM image of a fabricated micro-disk laser on Si with a diameter of 4 μm.

Figure 5a,b show the schematic diagram and scanning electron microscopy (SEM) image of the silicon-based micro-disk laser with a diameter of 4 μm by using the sample as shown in Figure 1, which contains 7 layers of InAs/InGaAs QDs as the active region for high optical gain.

The micro-disk structure was fabricated by a two-step etching method [16]. First, the silica beads with diameter of 4 μm were scattered on the surface of the sample, acting as the hard mask for mesa etching. After dry etching by ICP (inductively coupled plasma) process, the sample with pillar mesas was dipped in a 50% diluted HCl solution for selective wet etching of 600 nm InGaAlAs sacrificial layers to construct supporting pillar as shown in Figure 5a. The detailed fabrication process can be referred to in our previous work [16]. Figure 5b shows the SEM image of a fabricated disk laser on Si substrate, which presents a clean and smooth sidewall of the disk. By optimizing the wet etching process, a slender supporting pillar can be achieved, which can ensure the high optical confinement of the micro-disk.

## 3. Results and Discussions

**Figure 6:**
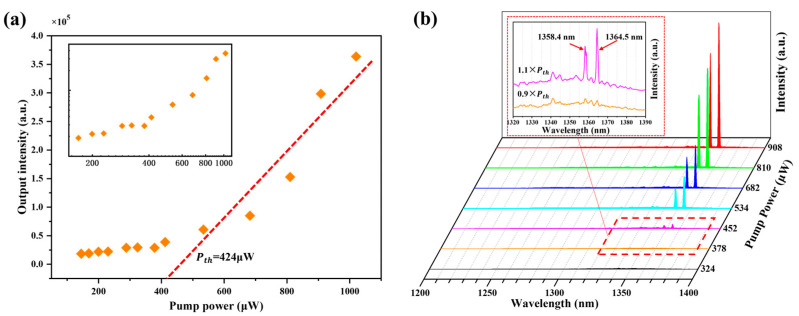
(**a**) The output intensity of the micro-disk laser on Si substrate versus the effective pumping power (L-L curve). Inset: the log-log scale plot of the non-linear L-L curve. (**b**) Pumping-power-dependent lasing spectra of the laser device. Inset: zoomed-in spectra collected at the pumping power below and above threshold.

For characterizations of the device properties, the micro-disk laser sample was mounted in liquid-helium-cooled cryostat and measured under a micro-photoluminescent (μPL) system with a CW 532 nm solid-state pumping laser. All the optical properties of the laser were characterized at a temperature of 200 K. Figure 6a,b show the relationship of the laser output intensity as a function of pumping power (L-L curve) and the power-dependent emission spectra of the laser, respectively.

The orange square symbols in Figure 6a represent the experimental data, fitted by a red dotted line. Here, the threshold power of this micro-disk laser on Si is calculated as a value of 0.424 mW (Standard error: 0.057) from the L-L curve. The inset of Figure 6a presents the log-log scale plot of the non-linear L-L curve, showing an “S” shape, which demonstrates three modes of nonlinear transition of lasing phenomena: spontaneous emission, amplified spontaneous emission and laser oscillation [32]. Figure 6b shows the spectral evolution of the micro-disk laser with an increase in the pumping power from 0.324 mW to 0.908 mW. At a low pumping power, broad spontaneous emission spectra were observed. As the pumping power gradually increased, obvious sharp peaks of the micro-disk laser on the Si substrate occurred. The inset of Figure 6b shows the zoomed-in spectra of the laser below (0.9 × P_th_) and above (1.1 × P_th_) the threshold power, displaying that the dominant lasing wavelength peaked at 1364.5 nm and another one at 1358.4 nm. Here, an obvious blue shift in the lasing peak (1364.5 nm) can be found referring to the RT PL spectrum of the InAs QDs on Si (peak: 1434.7 nm), which is caused by the typical temperature dependence of the peak position for semiconductor materials [33,34].

**Figure 7:**
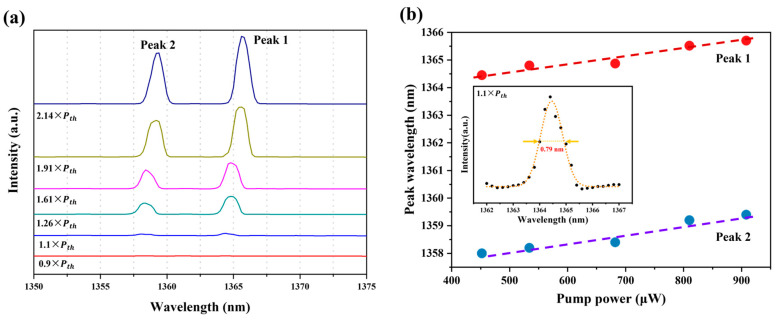
(**a**) Zoomed-in spectra of the micro-disk laser on the Si substrate at the increasing pumping power, indicating multi-peak lasing property. (**b**) The relationship of the two lasing peak wavelengths as a function of the pumping power. Inset: zoomed-in spectrum of the dominant peak (1364.5 nm) from the laser fitting by Lorentzian curve at 1.1 × P_th_, showing a 0.79 nm linewidth.

Figure 7a indicates the zoomed-in spectra of the Si-based micro-disk laser at increasing pumping power, which shows an obvious red shift in the two peak wavelengths due to the thermal effects from a pumping laser.

Figure 7b shows the relationship of the two lasing peak wavelengths as a function of the pumping powers. The red circle symbols display the experimental data of dominant emission peak (Peak 1), extracted from Figure 7a. The shifting rate of Peak 1 is calculated with a value of 2.69 nm/mW (Standard error: 0.39) from the fitting results in Figure 7b, which is slightly smaller than that (3.2 nm/mW with standard error of 0.46) of Peak 2. The inset of Figure 7b displays the zoomed-in Peak 1 spectrum of the micro-disk laser on Si at the pumping power slightly above the threshold power (1.1 × P_th_), and the experimental data are fitted by a Lorentzian fitting line shape (orange curve). Here, the cavity quality factor (Q) of the micro-disk can be calculated with Q $=\lambda_{cav}/{\Delta \lambda }_{cav}$, in which ${\Delta \lambda }_{cav}$ is the linewidth with a value of 0.79 nm obtained from Lorentzian fitting curve and $\lambda_{cav}$ is the dominant lasing peak of 1364.5 nm. The Q factor of the E-band micro-disk laser on Si (001) substrate is obtained as a value of 1727.2, which is a little higher than that (1626) of the S-band micro-disk laser on SOI substrates [16]. This is probably due to the lower defect density of the In_0.25_Ga_0.75_As on GaAs/Si (001) substrates used here than that of In_0.35_Ga_0.65_As on the GaAs/SOI platforms for S-band lasing.

## 4. Conclusions

In summary, we have successfully demonstrated the optically pumped, direct epitaxially grown E-band InAs QD micro-disk lasers on GaAs/Si (001) substrates by using the ${In}_{0.25}{Ga}_{0.75}As$ metamorphic buffer layer. The crystal quality and optical properties of the E-band InAs/InGaAs QDs on Si (001) are investigated and discussed. The fabricated micro-disk laser with a 4 µm diameter presents a low threshold power of 0.424 mW and a Q factor of 1727.2 at 200 K. In order to further enhance the performance of the device, our forthcoming efforts will concentrate on optimizing the epitaxial growth parameters and structures, aiming to diminish the dislocation densities and improve the crystal quality. Overall, the results presented here provide a promising solution to realize on-chip telecom light sources not only for silicon photonic integration but also for potential sensing applications.

## Data Availability

Data are contained within the article.
